# Behavioural tasks sensitive to acute abstinence and predictive of smoking cessation success: a systematic review and meta‐analysis

**DOI:** 10.1111/add.13507

**Published:** 2016-08-08

**Authors:** Meryem Grabski, H. Valerie Curran, David J. Nutt, Stephen M. Husbands, Tom P. Freeman, Meg Fluharty, Marcus R. Munafò

**Affiliations:** ^1^ School of Experimental PsychologyUniversity of BristolBristolUK; ^2^ MRC Integrative Epidemiology Unit (IEU) at the University of BristolBristolUK; ^3^ UK Centre for Tobacco and Alcohol StudiesUniversity of BristolUK; ^4^ Clinical Psychopharmacology UnitUniversity College LondonLondonUK; ^5^ Department of MedicineImperial College LondonLondonUK; ^6^ Department of Pharmacy and PharmacologyUniversity of BathBathUK

**Keywords:** Abstinence, cessation, cognition, meta‐analysis, performance, smoking, systematic review, tobacco

## Abstract

**Background and aims:**

Performance on cognitive tasks may be sensitive to acute smoking abstinence and may also predict whether quit attempts fail. Our aim was to conduct a systematic review and meta‐analysis to identify cognitive tasks sensitive to acute abstinence and predictive of smoking cessation success.

**Methods:**

Embase, Medline, PsycInfo and Web of Science were searched up to March 2016. Studies were included if they enrolled adults and assessed smoking using a quantitative measure. Studies were combined in a random effects meta‐analysis.

**Results:**

We included 42 acute abstinence studies and 13 cessation studies. There was evidence for an effect of abstinence on delay discounting [d = 0.26, 95% confidence interval (CI) = 0.07–0.45, *P* = 0.005], response inhibition (d = 0.48, 95% CI = 0.26–0.70, *P* < 0.001), mental arithmetic (d = 0.38, 95% CI = 0.06–0.70, *P* = 0.018), and recognition memory (d = 0.46, 95% CI = 0.23–0.70, *P* < 0.001). In contrast, performance on the Stroop (d = 0 .17, 95% CI = −0.17–0.51, *P* = 0.333) and smoking Stroop (d = 0.03, 95% CI = −0.11–0.17, *P* = 0.675) task was not influenced by abstinence. We found only weak evidence for an effect of acute abstinence on dot probe task performance (d = 0.15, 95% CI = −0.01–0.32, *P* = 0.072). The design of the cessation studies was too heterogeneous to permit meta‐analysis.

**Conclusions:**

Compared with satiated smokers, acutely abstinent smokers display higher delay discounting, lower response inhibition, impaired arithmetic and recognition memory performance. However, reaction‐time measures of cognitive bias appear to be unaffected by acute tobacco abstinence. Conclusions about cognitive tasks that predict smoking cessation success were limited by methodological inconsistencies.

## Introduction

In economically developed countries cigarette smoking is the greatest preventable cause of death, and world‐wide more than 5 million people die prematurely each year from this 1. However, despite the fact that most smokers are aware of the risk smoking poses to their health, many fail to quit permanently. One important factor in the continuation of smoking behaviour and the occurrence of relapse is the withdrawal state 2, 3. Consequently, many effective smoking cessation methods currently available target the management of adverse withdrawal symptoms 4, 5. Despite this, only approximately 20% of smokers using one of these methods are successful in achieving long‐term abstinence 6.

A clearer understanding of the underlying mechanisms of withdrawal is vital in order to develop novel behavioural and pharmacological treatments for smoking cessation. One way of facilitating the discovery of treatments is to create a behavioural model of withdrawal in humans. Here, a behavioural outcome that is linked to withdrawal symptoms can act as a marker to indicate the efficacy of a treatment in a time‐ and cost‐effective way. Performance on behavioural laboratory tasks can be measured precisely and objectively, and is therefore less prone to bias than self‐report measures of withdrawal symptoms. Due to the fact that task performance has been linked to short‐term abstinence 7, 8, 9 and to long‐term cessation outcomes 10, 11, this might represent a behavioural marker for treatment development efforts.

Short‐term (or acute) abstinence is measured by subjecting participants to a set period during which they are prohibited from smoking. A comparison of performance after a period of acute abstinence to performance in a non‐abstinent state is used to indicate cognitive functions affected by tobacco withdrawal. Here these studies are referred to as ‘abstinence studies’. Long‐term cessation outcomes are measured by following‐up success rates of participants who are attempting to quit smoking. A measure of performance at the beginning of a quit attempt can thus be linked to relapse rates, and act as a potential predictor of smoking cessation success. These studies will be referred to as ‘cessation studies’. Several kinds of task have been investigated intensively in behavioural nicotine research: tasks measuring cognitive bias, cognitive performance and impulsivity.

Changes in cognitive biases towards drug‐related cues are believed to occur in dependent users of any centrally acting drug 12, 13. Cues associated with drugs of abuse are hypothesized to trigger a sensitized reward system and are therefore more likely to ‘grab’ the attention of the user, leading to craving and a higher risk of relapse 14, 15. Several laboratory tasks have been used to assess changes in cognitive bias in smokers. In the dot probe task participants have to make a response towards a probe that appears either behind a smoking‐related or a matched neutral stimulus. Faster responding to probes in the smoking‐stimulus location, relative to probes in the neutral‐stimulus location, is taken as evidence of cognitive bias toward smoking‐related stimuli. A related task is the smoking Stroop task, a modification of the classic colour‐naming Stroop task 16 in which participants are instructed to indicate the ink colour of a list of colour‐words, thereby suppressing the automatic tendency to read the semantic content of the words. In the smoking Stroop task, matched neutral and smoking‐related words are used. Selective processing is reflected in longer response times or more errors to smoking‐related than to neutral words. During tobacco withdrawal this selective processing is hypothesized to be enhanced. Even though some studies have found increased cognitive bias during acute abstinence, others have failed to do so 17, 18, 19. It has also been suggested that increased cognitive bias towards smoking‐related cues might be predictive of cessation outcomes 10.

Another avenue of research stems from the cognitive performance benefits many smokers report following acute nicotine consumption 20. During cessation, the absence of those benefits might reduce the likelihood of a successful quit attempt. A negative effect of abstinence on cognitive performance has been found in several studies investigating domains, such as mental arithmetic, working memory and continuous attention 21, 22, 23. Disrupted performance is reflected in higher error rates or longer reaction‐times in the performance of smokers in the abstinent compared to the satiated condition. It has been suggested that abstinence‐induced cognitive deficits might also be predictive of poor cessation outcomes 24.

A further domain of interest is impulsivity. Increased impulsivity has been related to drug use and difficulties with cessation 25. The ability to inhibit an unwanted response (i.e. response inhibition) is one aspect of impulsivity 26, 27. Abstinent smokers have been found to display less response inhibition on the ‘go/no‐go’ and ‘stop‐signal’ tasks, which require participants to inhibit a learned response towards a specific stimulus 28. Another aspect of impulsivity is the discounting of hypothetical future rewards, such as money. Higher discounting is characterized by a preference of smaller, more immediate rewards over larger, delayed rewards. Several studies have found higher delay discounting in abstinent as compared with satiated smokers 29, 30.

Considering the range of tasks used in the literature it is still unclear which tasks are best suited to reliably indicate brain changes in acute abstinence and predict cessation outcome. In the current review we were particularly interested in behavioural tasks that display both properties: sensitivity to acute abstinence, as well association with cessation outcomes, as both of these properties could be utilized to inform the development and refinement of treatments for smoking cessation. To our knowledge, no attempt has been made so far to systematically review the literature on these issues. We therefore conducted a systematic review examining the following questions regarding measures of cognitive bias and cognitive performance: (a) which tasks are most sensitive to acute abstinence in smokers and (b) which tasks are predictive of smoking cessation?

## Methods

### Search strategy

**Figure 1:**
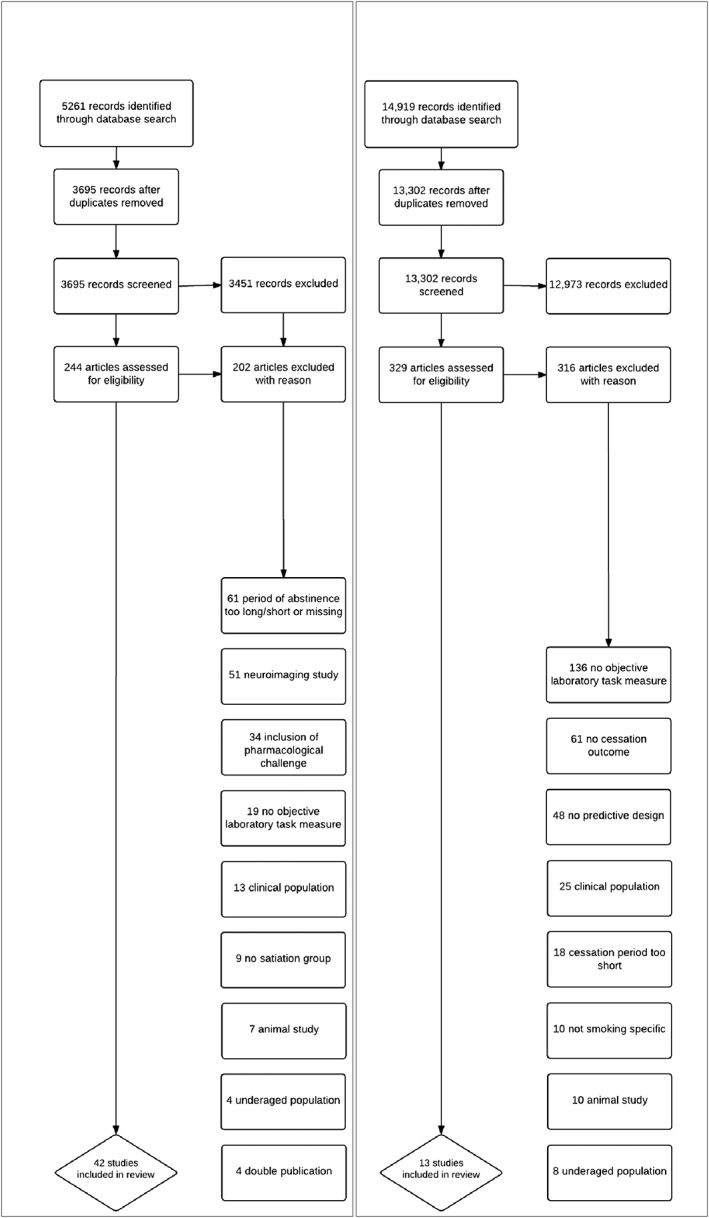
Flow diagram of study selection. Study selection flow‐chart shown for abstinence studies (left) and cessation studies (right)

The literature was searched using the electronic databases Embase, Medline, PsycInfo and Web of Science up to the beginning of March 2016. Searches were limited to peer‐reviewed papers written in English and involving humans. Boolean operators and truncations were modified slightly, depending on the database. We used the following general keywords for the search: cogn*, attention, executive function*, impuls*, memory, information process*, Stroop, CARROT, eye‐track*, emotion recognition, temporal discount*, delay discount*, risk taking, inhibit*, smok*, cigarette*, nicotine, tobacco. For abstinence studies we included the following additional keywords: abstinence, craving, deprivation, withdrawal. For cessation studies the following keywords were additionally included: outcome, result, predict*, marker, maintain*, success*. The full search strategy is available as online supporting information. Search results for acute abstinence and cessation studies are presented in Fig. 1. One of the authors (M.G.) reviewed the electronic titles and abstracts and selected the full‐text articles to be included. A 10% check of study inclusion was carried out independently by another author (M.F.), and agreement was 100%.

### Selection criteria

Studies were included in the review if they met the following general criteria: the use of at least one quantitative measure of smoking behaviour to characterize participants as smokers [e.g. cigarettes per day, years of continuous smoking, Fagerström Test for Nicotine Dependence score (FTND)^31^, etc.]; and samples comprising participants aged 18 years or older. Studies were excluded if psychiatric patient groups were investigated.

#### Abstinence studies

Specific selection criteria for abstinence studies were: comparison of smokers’ task performance in satiated and deprived states; assessment of task performance by one or more laboratory behavioural tasks; and biochemically confirmed overnight‐abstinence before testing for the deprived state (a minimum of 8 hours was chosen to capture overnight abstinence sufficiently). Abstinence studies were excluded if any form of nicotine or other pharmacological challenge was given before testing. We refrained from including neuroimaging studies as we considered their methods to be too different to other laboratory methods to be comparable.

#### Cessation studies

Specific selection criteria for cessation studies were: studies following‐up cessation attempts of smokers up to a minimum of 1 month; assessment of task performance by one or more behavioural tasks before or at the beginning of a cessation attempt; and precise description of support/cognitive behavioural therapy/medication given during cessation attempt. The last criterion was included to account for possible differential effects of these treatments on the relationship between task performance and cessation outcome.

### Data extraction

The following information was coded for each of the studies: tasks used; outcome measures; source statistic used to calculate effect sizes; mean age of participants; and sex distribution of participants. Further information, if available, included: mean baseline FTND score and mean baseline exhaled carbon monoxide (CO) level. Specific information coded for the abstinence studies included: study design (within‐ or between‐subjects); length of deprivation period and quantitative measure of smoking behaviour. Specific information coded for the cessation studies included: time‐points of follow‐up; nature of groups compared (treatment groups, placebo groups, etc.); number of participants at first and last assessment point, smoking status at baseline assessment and nature of treatment, if applicable.

The data extraction was carried out by M.G. and a subset of 10% of studies were extracted independently by M.F. in order to check for error and bias. Agreement between the raters was acceptable (> 90%), and discrepancies were resolved by mutual consent. The source statistics of all the papers for which effect sizes were reported were coded by M.G. and independently checked by M.F., and agreement was 100%.

Effect sizes were calculated for all the tasks for which appropriate source statistics were available, or where these could be obtained from the authors. In cases where several statistics were available, the calculation of effect sizes from descriptive statistics was preferred over their calculation from test statistics. For the calculation of the effect sizes the methods described by Cooper *et al*. 32 were used.

The analysis of the response inhibition tasks included two stop‐signal tasks 27, 33, as well as a go/no‐go task 28. We considered the underlying construct captured by both tasks to be sufficiently similar to warrant their synthesis in a meta‐analysis.

### Data analysis

A meta‐analysis was conducted on every task that was reported by a minimum of three studies and where outcome measures were identical (e.g. all reaction‐times or all error rates). This resulted in the analysis of the following seven abstinence tasks: (1) delay discounting tasks, (2) response inhibition tasks, (3) mental arithmetic tasks, (4) recognition memory tasks, (5) Stroop tasks, (6) smoking Stroop tasks and (7) dot probe tasks. We conducted a sensitivity analysis in order to determine whether the inclusion of the go/no‐go task changed the results of the response inhibition task analysis substantially by running the meta‐analysis with and without the go/no‐go task included. Due to the similarity of the underlying constructs investigated, we conducted a sensitivity analysis to determine whether combining the smoking Stroop and the dot probe task would have provided different results. Details on tasks included in the analyses are summarized in Table 1

**Table 1:** Studies included in the meta‐analyses.

*Task*	*Task description*	*Name*	*Year*	*n*	*Mean age (years)*	*Sex (% f)*	*Abstinence period (hours)*	*Mean FTND*	*Minimum cig/day*	*Study design*	*Outcome measure*		*Effect size d*
Delay Discounting	Participants indicate their preference of hypothetical rewards at different delay points. A preference of smaller, sooner rewards over higher, more long‐term rewards is believed to indicate higher impulsivity. A positive effect size d reflects higher delay discounting in abstinent than in satiated smokers.	Ashare	2012	56	40	52%	14	5.3	10	W	Discount rates		0.09
		Ashare	2015	33	39	36%	24	4.7	10	W	Discount rates		0.08
		Field	2006	31	23	48%	13	3.6	10	W	Discount rates		0.46
		Mitchell	2004	11	20	45%	24	5.0	15	W	Discount Rates		0.11
		Roewer	2015	37	33	41%	24	7.2	25	W	Discount rates		0.25
		Yi	2012	28	40	28%	24	6.4	20	W	Discount rates		0.65
Response Inhibition	Participants indicate whether a stimulus is a go or a no‐go (or stop) stimulus. The response to the go‐stimulus is prepotent. Greater difficulty to switch to the no‐go (or stop) response is thought to indicate higher impulsivity. A positive effect size d reflects less response inhibition in abstinent than in satiated smokers.	Ashare	2012	56	40	52%	14	5.3	10	W	SST RTs		0.34
		Charles‐Walsh	2014	22	26	50%	10	–	15	W	SST RTs		0.70
		Harrison	2009	30	32	50%	17	5.1	10	W	GNG RTs		0.43
Mental Arithmetic	Participants have to solve arithmetic tasks mentally. A positive effect size d reflects higher error rates in abstinent than in satiated smokers.	Al'Absi	2002	30	25	50%	18	5.3	15	W	Error rates		0.39
		Elgerot	1976	12	–	33%	15	–	15	W	Error rates		0.88
		Leventhal	2010	195	36	50%	12	6.5	15	W	Error rates		0.17
		Parrott	1998	30	25	53%	12	–	10	B	Error rates		0.22
Recognition Memory	Participants have to indicate words on a list that they have been shown previously. A positive effect size reflects more impaired working memory performance in abstinent than in satiated smokers.	Hirshman	2004	20	23	45%	24	5.9	–	W	Memory discrimination ‘d’		0.60
		Merritt	2010	25	–	52%	24	–	–	W	Memory discrimination ‘d’		0.38
		Merritt	2012	25	23	52%	24	4.4	–	W	Memory discrimination ‘d’		0.43
Stroop	Participants are instructed to indicate the ink colour of a list of colour‐ words. A positive effect size d reflects higher interference scores in abstinent than in satiated smokers.	Domier	2007	43	37	42%	13	5.1	15	W	Bias scores (RTs)		0.02
		Mogg	2002	27	33	51%	12	3.9	10	W	Bias scores (RTs)		0.02
		Pomerleau	1994	13	28	100%	12	5.9	15	W	Bias scores (RTs)		0.66
Smoking Stroop	Participants are instructed to indicate the ink colour of smoking related and matched neutral words. A positive effect size reflects stronger cognitive bias in abstinent than in satiated smokers.	Canamar	2012	51	37	37%	13	5.3	15	W	Bias scores (RTs)		<0.01
		Leventhal	2010	195	36	50%	12	6.5	15	W	Bias scores (RTs)		0.05
		Mogg	2002	27	33	51%	12	3.9	10	W	Bias scores (RTs)		<0.01
		Munafò	2003	43	28	51%	24	2.2	1	B	Bias Scores (RTs)		0.39
		Waters	2000	24	–	–	24	–	7	B	Bias scores (RTs)		−0.90
Dot Probe	Participants have to respond manually to a probe, appearing either after presentation of a smoking‐related or a neutral image. A positive effect size reflects stronger cognitive bias in abstinent than in satiated smokers.	Freeman	2012	48	27	40%	12	4.7	10	B	Bias scores (RTs)		0.32
		Leventhal	2010	100	36	50%	12	6.5	15	W	Bias scores (RTs)		0.17
		Mogg	2002	27	33	51%	12	3.9	10	W	Bias scores (RTs)		0.01

FTND = Fagerström Test of Nicotine Dependence; RTs = reaction‐times; GNG = go/no‐go task; SST = stop signal task.

The R software package ‘metafor’ 34 was used to analyse the data. We chose a random‐effects model in order to account for the possibility of high heterogeneity between studies 35. In the absence of between‐study heterogeneity, a random‐effects model gives the same results as a fixed‐effects model. In order to assess between‐study heterogeneity we calculated the *I* ^2^ statistic 36. A value of *I* ^2^ is regarded conventionally as low at 25%, medium at 50% and high at 75%.

Where the correlation between the two test measures was required for the calculation of effect sizes (e.g. within‐subjects designs) we set this at *r* = 0.5, as this information was not provided in the individual studies. A sensitivity analysis was conducted, setting *r* at different levels (*r* = 0.2, *r* = 0.8), in order to establish whether the results of the meta‐analysis were robust to varying this assumption.

## Results

### Abstinence studies

A total of 42 studies met the inclusion criteria and were retained in the final sample (Supporting information, Table S1). The aggregate sample size of the included studies was 1758 participants. The majority of studies was somewhat small, with 58% of the studies including 30 or fewer participants [mean = 43; median = 27; interquartile range (IQR) = 23]. Most studies were relatively recent, with only nine studies published before 2000. The studies were predominantly randomized within‐subject designs, where smokers were tested once while abstinent and once while non‐abstinent. Eight studies employed a between‐subjects design, which compared performance of a group of abstinent smokers to performance of a group of non‐abstinent smokers.

The participant population was comprised mainly of young adults: the mean age, weighted by the sample size of the studies that reported it (*k* = 34), was 33 years. Approximately 45% of all participants were female (*k* = 38). A weighted mean FTND score of 5.1 indicated moderate to high nicotine dependence (*k* = 27). The average abstinence period of all studies was 18 hours. Twenty‐three studies reported baseline exhaled CO levels, which had a weighted average of 25.1 parts per million (p.p.m.). The following tasks were reported by three or more studies, and therefore meta‐analysed: delay discounting 29, 30, 33, 37, 38, 39, response inhibition 27, 28, 33, mental arithmetic 23, 40, 41, 42, recognition memory 43, 44, 45, Stroop 46, 47, 48, smoking Stroop 19, 41, 47, 49, 50 and dot probe tasks 41, 47, 51. As the number of studies in each analysis was relatively small (*k* = 3–6), we did not explore study‐level moderators.

Meta‐analyses of the delay discounting, response inhibition, mental arithmetic and recognition memory tasks indicated evidence of an effect of acute abstinence. A sensitivity analysis indicated that including the go/no‐go task in the analysis of response inhibition tasks did not change the result substantially. Results for the meta‐analysis of the dot probe task indicated only weak evidence of an effect of acute abstinence on cognitive bias. Furthermore, there was no evidence of an effect of acute abstinence on the Stroop and smoking Stroop tasks. A sensitivity analysis indicated that combining both cognitive bias tasks (dot probe and smoking Stroop) did not result in a different pattern of results. These results are summarized in Table 2.

**Table 2:** Meta‐analysis results.

*Task*	*k*	*N*	*d*	*95% CI*	*P‐value*	*I* ^*2*^
Delay discounting	6	196	0.26	0.07 to 0.45	0.005	35%
Response inhibition	3	108	0.48	0.26 to 0.70	< 0.001	28%
Mental arithmetic	4	267	0.38	0.06 to 0.70	0.018	67%
Recognition memory	3	70	0.46	0.23 to 0.70	< 0.001	0%
Stroop	3	83	0.17	−0.17 to 0.51	0.333	56%
Smoking Stroop	5	340	0.03	−0.11 to 0.17	0.675	0%
Dot probe	3	175	0.15	−0.01 to 0.32	0.072	0%

K = number of studies included in analysis, *N* = aggregate number of participants, d = effect size, 95% CI = confidence interval of effect size, *I* ^2^: between‐study heterogeneity.

A sensitivity analysis of the within‐subject design studies revealed that setting the correlation between the two measures at different values (*r* = 0.2, *r* = 0.8) did not change the results of our meta‐analysis substantially.

We also performed a risk of bias assessment as recommended by the Cochrane Collaboration for all studies included in the meta‐analysis. For the vast majority of measures of bias there was insufficient information to rate studies as anything other than ‘unclear’. The exception was attrition, where four studies were rated as having a high risk of bias, of which two contributed to the analysis of recognition memory 43, 44, one to the analysis of the Stroop task 46 and one to the analysis of the smoking Stroop task 50. Another three studies, contributing to the meta‐analyses of mental arithmetic 23, delay discounting 33 and dot probe tasks 51 were rated as having a low risk of bias.

### Cessation studies

Thirteen studies were included (Supporting information, Table S2). The aggregate sample size of the included studies was 2081 participants. Two studies had fewer than 20 participants and seven studies 100 or more participants in total at baseline (mean = 160; median = 97 ; IQR = 134). Designs varied considerably, with follow‐up ranging from 1 to 24 months. The weighted average age of the participants was 44 years (*k* = 13). The sex distribution was roughly even, with 56% female participants (*k* = 13). Two studies included pharmacological treatments only 52, 53, one study included psychological treatments only 54 and four studies included pharmacological as well as psychological treatments 55, 56, 57, 58; the remaining five studies did not include any treatment 59, 60, 61, 62, 63.

Seven tasks were associated significantly with smoking cessation success at the study end‐point. Two measures of impulsivity, the delay discounting task and a discrete choice task, were found to predict cessation at 4, 5 and 6 months 57, 60, 63. Powell and colleagues 62 reported motor impulsiveness to predict cessation success at 3 months. Motor impulsiveness was assessed with a continuous performance task, in which participants had to indicate the occurrence of two consecutive targets via button‐press. Furthermore, several measures of attention, such as the Simon task 53, in which executive control during exposure to affective, neutral and smoking stimuli was measured, and a startle response task 61, in which eyeblink‐amplitude was measured after presentation of an acoustic startle stimulus, were reported to predict cessation at 2 and 1 months, respectively. The design of the studies was too heterogeneous to allow for meta‐analyses.

## Discussion

Meta‐analyses were conducted on all tasks that were reported by three or more studies, resulting in the analyses of seven types of tasks from acute abstinence studies. The analysis of the delay discounting task indicated that short‐term, smaller rewards were preferred over long‐term, larger rewards, indicating higher impulsivity in abstinent smokers. Rewards used in these studies were hypothetical gains of cigarettes or money. Due to the small number of studies included, it was not possible to explore these types of rewards differentially. Another task where there was good evidence for an effect of acute abstinence on performance was the response inhibition task, which is considered generally a measure of impulsivity. Impaired response inhibition is theorized to reflect the inability to prevent behaviours with negative consequences 64; in the context of tobacco abstinence, this equates to the constant need to inhibit the pre‐potent tendency to smoke. This was reflected in our findings, where abstinent smokers had more difficulty in inhibiting a pre‐potent response than satiated smokers. The other two tasks that indicated good evidence for an effect of acute abstinence on performance were the recognition memory and mental arithmetic tasks. For both tasks, participants in the acute abstinence condition performed worse than in the satiated condition, but the interpretation of these performance decrements is less straightforward. One common process underlying both tasks might be the engagement of working memory, so these results could reflect diminished working memory performance. However, it is equally possible that a more general decrement of cognitive abilities is driving these results, as not enough domains have been analysed in order to specify the abilities most affected.

Those tasks designed specifically to assess cognitive bias towards smoking‐related cues were not found to be affected by acute abstinence. There was only weak evidence for the dot probe tasks showing an effect of abstinence. Furthermore, there was no evidence for the smoking Stroop or the normal Stroop tasks being influenced by acute abstinence. One explanation might be the methods used to investigate cognitive bias. Several studies have suggested that, despite the finding that smokers show a cognitive bias towards smoking related cues compared to non‐smokers, there is no difference between satiated and deprived smokers 19, 47, 65. This could be due to the fact that such a short period of abstinence does not lead to a pronounced change in cognitive bias, or that reaction‐time tasks might not be sensitive enough to these subtle changes.

Because the cessation studies varied considerably in the use of pharmacological and behavioural challenges, the time‐points of follow‐up and the tasks used for assessment, it was not possible to quantitatively synthesize their results. Nevertheless, several studies reported that different cognitive measures were predictive of smoking abstinence, such as measures of impulsivity 57, 60, 62, 63, cue–reactivity 62 and attention 53, 61.

Our analysis suggests that cognitive bias reaction‐time tasks might be unsuitable because they appear to be uninfluenced by acute abstinence. A recommendation for which tasks to use is more difficult, as the tasks analysed were too few and too varied to draw any strong conclusions. Further research is needed, focusing potentially on working memory and impulsivity as a phenotype for treatment developments. More importantly, future research should consider recruiting larger sample sizes and replicating earlier findings, as it was difficult to draw definite conclusions because of methodological differences between studies. Such an approach would enable the development of a battery of tasks known to be sensitive to acute abstinence. Employing established cognitive tasks alongside novel measures could also act as a positive control (i.e. manipulation check).

There are several limitations to the conclusions drawn from the meta‐analyses. First, the majority of the studies analysed was relatively small, with 65% of studies having a sample size of 30 or fewer participants; only one study included more than 100 participants 41. Secondly, between‐study heterogeneity was moderate to high for two of the tasks that showed an effect of acute abstinence on cognition (mental arithmetic and delay discounting). This casts some doubt on the reliability of the observed effect sizes for these tasks. Nevertheless, as the number of studies included in all the meta‐analyses was small, the estimation of variance should be interpreted carefully, as a small number of studies tends to decrease strongly the power of the estimators of variability 34. Thirdly, due to the small number of studies, we were also not able to test for publication bias. As it is likely that some studies with negative results have not been published, there is a risk of an inflation of our effect size estimates. Fourthly, a lack of information made it difficult to rate the risk of bias of most studies included in the meta‐analyses. Fifthly, all the measures that did not exhibit evidence of sensitivity to acute abstinence were difference scores. As difference scores are more likely to suffer from low reliability 66, a smaller effect of abstinence on these measures might be expected due to this alone. Thus, our finding that these measures were less sensitive to acute abstinence should be interpreted carefully. Sixthly, the analysis of the response inhibition tasks included two types of tasks, the go/no‐go and stop‐signal tasks. The main difference between these is that the former requires a decision to either inhibit a response towards a no‐go cue or to execute a response towards a go‐cue, whereas in the latter the stop signal occurs only after the go signal has already been given, thus requiring the participant to change into a stop decision after a go decision has already been made. In a sense, therefore, the stop‐signal task requires a higher load on response inhibition processes, and may be considered more difficult in this respect than the go/no‐go task. However, a stable effect of acute abstinence on response inhibition was found when the go/no‐go task was omitted from analysis. We therefore suggest that in the context of the acute abstinent paradigm these two tasks are similar enough to be synthesized in a meta‐analysis. Similarly, we analysed two measures of cognitive bias (smoking Stroop and dot probe tasks) separately. In order to ensure that our results were not influenced by our decision to group these tasks in this way, we conducted a meta‐analysis in which smoking Stroop and dot probe tasks were combined. This result was not substantially different from our original findings. Seventhly, the acute abstinence paradigm does not allow for an investigation on whether the changes in task performance are actually due to withdrawal or, instead, what Hughes has described as the ‘offset effect’ 67 (i.e. sustained changes following drug cessation, rather than the transient effects of withdrawal). Whether or not the effects of abstinence on cognition are transient is difficult to determine in a short‐term study, such as the acute abstinence paradigm. Nevertheless, whether the changes in cognition observed are a direct result of withdrawal or an offset effect should not affect the value of our findings as they could both drive relapse, and could therefore act as an indicator of treatment results 68.

In conclusion, our meta‐analyses revealed no strong evidence of an effect of acute abstinence on cognitive bias reaction‐time tasks. Conversely, delay discounting, response inhibition, recognition memory and mental arithmetic tasks were found to be sensitive to acute abstinence. The systematic review of studies investigating tasks predictive of long‐term smoking cessation outcome indicated that research on this topic is currently lacking.

## Declaration of interests

M.G. is funded by an Economic and Social Research Council PhD studentship with co‐funding from the pharmaceutical company Rusan Pharma Ltd. H.V.C. receives funding from the UK Medical Research Council. D.J.N. has received research funding from P1Vital, as well as share options. He has acted on the advisory boards of the following pharmaceutical companies: Lundbeck, Servier, Pfizer, Reckitt Benkiser, D&A Pharma, Novartis, MSD, Nalpharm and Actelion, and received additional speaking honoraria from: Bristol‐Myers Squibb, GlaxoSmithKline, Schering‐Plough, Lilly and Janssen. He has acted as an adviser to the British National Formulary, the Department of Health, the Medical Research Council and the Swedish Government, for the latter specifically on drug, alcohol and tobacco research. He is a member of the Lundbeck International Neuroscience Foundation and the International Centre for Science in Drug Policy. He is furthermore the director of Equasy Enterprises Ltd and Chaperon Ltd. S.M.H. receives funding from the National Institute on Drug Abuse (NIH). M.R.M. has received research funding from various research councils and charities, which have included the Alcohol Education and Research Council, the European Research Advisory Board and Pfizer. In addition, he has received nicotine replacement products from GlaxoSmithKline and Pfizer for distribution to study participants.

## Supporting information


**Table S1** Search 1 study characteristics.

**Table S2** Search 2 study characteristics.
