# Supplementary material for: Behavioural tasks sensitive to acute abstinence and predictive of smoking cessation success: a systematic review and meta‐analysis
Source: Addiction. 2016 Aug 8;111(12):2134–44. doi: 10.1111/add.13507 (PMC5111768; doi:10.1111/add.13507)
Supplement: Supplementary file 1 — Table S1 Search 1 study characteristics. [file ADD-111-2134-s001.docx]

**Supplementary Table 1. Search 1 study characteristics**

|  | **Name** | **Year** | **N** | **Mean Age (yrs)** | **Sex (% f)** | **Abstinence Period (hrs)** | **Mean FTND** | **Minimum Cig/ Day** | **Mean Baseline CO (ppm)** | **Cognitive Tasks** | **Effect Size d*** | **Variance** |
| --- | --- | --- | --- | --- | --- | --- | --- | --- | --- | --- | --- | --- |
| 1 | Al’ Absi | 2002 | 30 | 25 | 50% | 18 | 5.3 | 15 | 19.0 | Mental Arithmetic | 0.39 | 0.04 |
|  |  |  |  |  |  |  |  |  |  | Paced Auditory Serial Addition | 0.61 | 0.04 |
| 2 | Ashare | 2012 | 56 | 40 | 52% | 14 | 5.3 | 10 | 34.5 | Delay Discounting | 0.09 | 0.02 |
|  |  |  |  |  |  |  |  |  |  | Startle Continuous Performance | 0.46 | 0.02 |
|  |  |  |  |  |  |  |  |  |  | Stop Signal | 0.34 | 0.02 |
| 3 | Ashare | 2015 | 33 | 39 | 36% | 24 | 4.7 | 10 | 19.4 | Delay Discounting | 0.08 | 0.03 |
| 4 | Canamar | 2012 | 51 | 37 | 37% | 13 | 5.3 | 15 | 24.3 | Smoking Stroop | <0.01 | 0.02 |
| 5 | Charles-Walsh | 2014 | 22 | 26 | 50% | 10 | - | 15 | 26.2 | Stop Signal | 0.70 | 0.03 |
| 6 | Domier | 2007 | 43 | 37 | 42% | 13 | 5.1 | 15 | 24 | Stroop | 0.02 | 0.02 |
| 7 | Elgerot | 1976 | 12 | - | 33% | 15 | - | 15 | - | Letter Series | 0.40 | 0.05 |
|  |  |  |  |  |  |  |  |  |  | Mental Arithmetic | 0.88 | 0.06 |
|  |  |  |  |  |  |  |  |  |  | Raven’s Progressive Matrices | 0.50 | 0.05 |
| 8 | Field | 2004 | 23 | 22 | 43% | 10 | 3.8 | - | 17.2 | Dot Probe | - | - |
| 9 | Field | 2006 | 31 | 23 | 48% | 13 | 3.6 | 10 | - | Delay Discounting | 0.46 | 0.04 |
|  |  |  |  |  |  |  |  |  |  | Behavioural Economics | - | - |
|  |  |  |  |  |  |  |  |  |  | Gambling | - | - |
| 10 | Freeman | 2012 | 48 | 27 | 40% | 12 | 4.7 | 10 | - | Drug Cue Reward Prediction Error | - | - |
|  |  |  |  |  |  |  |  |  |  | Dot Probe | 0.32 | 0.08 |
|  |  |  |  |  |  |  |  |  |  |  |  |  |
|  |  |  |  |  |  |  |  |  |  |  |  |  |
|  | **Name** | **Year** | **N** | **Mean Age (yrs)** | **Sex (% f)** | **Abstinence Period (hrs)** | **Mean FTND** | **Minimum Cig/ Day** | **Mean Baseline CO (ppm)** | **Cognitive Tasks** | **Effect Size d*** | **Variance** |
| 11 | Greenstein | 2009 | 23 | 30 | 0% | 12 | 4.4 | 10 | 13.0 | N-back | - | - |
|  |  |  |  |  |  |  |  |  |  | O-span | -0.48 | 0.04 |
|  |  |  |  |  |  |  |  |  |  | S-span | - | - |
| 12 | Harrison | 2009 | 30 | 32 | 50% | 17 | 5.1 | 10 | 25.0 | Connor’s Continuous Performance | 0.47 | 0.04 |
|  |  |  |  |  |  |  |  |  |  | Go/no-go | 0.44 | 0.04 |
| 13 | Hatsukami | 1989 | 19 | 22 | 53% | 24 | - | 10 | - | Digit Span | -0.79 | 0.13 |
|  |  |  |  |  |  |  |  |  |  | Trial Making | 0.85 | 0.14 |
|  |  |  |  |  |  |  |  |  |  | Symbol Digit Modalities | -0.27 | 0.10 |
|  |  |  |  |  |  |  |  |  |  | Vigilance | -0.18 | 0.10 |
| 14 | Havermans | 2003 | 36 | 23 | - | 12 | 4.1 | 10 | - | Digit Span | - | - |
|  |  |  |  |  |  |  |  |  |  | Sternberg | - | - |
| 15 | Heinz | 2007 | 60 | 34 | 50% | 12 | 5.7 | 15 | - | Rapid Serial Visual Attention | - | - |
| 16 | Hirshman | 2004 | 20 | 23 | 45% | 24 | 5.9 | - | 17.0 | Digit Span | - | - |
|  |  |  |  |  |  |  |  |  |  | Recognition Memory | 0.60 | 0.05 |
|  |  |  |  |  |  |  |  |  |  | Visual Attention | - | - |
| 17 | Hughes | 1989 | 16 | - | 0% | 24 | - | 20 | - | Digit Span | - | - |
|  |  |  |  |  |  |  |  |  |  | Sustained Attention | - | - |
|  |  |  |  |  |  |  |  |  |  | Visual Attentional Vigilance | - | - |
| 18 | Leventhal | 2008 | 113 | 24 | 62% | 12 | 3.8 | 10 | - | Subliminal Priming Smoking Stimuli | -0.26 | 0.02 |
|  |  |  |  |  |  |  |  |  |  |  |  |  |
|  |  |  |  |  |  |  |  |  |  |  |  |  |
|  | **Name** | **Year** | **N** | **Mean Age (yrs)** | **Sex (% f)** | **Abstinence Period (hrs)** | **Mean FTND** | **Minimum Cig/ Day** | **Mean Baseline CO (ppm)** | **Cognitive Tasks** | **Effect Size d*** | **Variance** |
| 19 | Leventhal | 2010 | 203 | 36 | 50% | 12 | 6.5 | 15 | 30 | Digit Symbol Substitution | -0.13 | <0.01 |
|  |  |  |  |  |  |  |  |  |  | Dot Probe | 0.17 | <0.01 |
|  |  |  |  |  |  |  |  |  |  | Rapid Information Processing | 0.25 | <0.01 |
|  |  |  |  |  |  |  |  |  |  | Mental Arithmetic | 0.17 | <0.01 |
|  |  |  |  |  |  |  |  |  |  | Smoking Stroop | 0.05 | <0.01 |
|  |  |  |  |  |  |  |  |  |  | Two- letter Search | 0.45 | <0.01 |
| 20 | Leventhal | 2012 | 75 | 42 | 28% | 18 | 5.3 | 10 | 29 | Emotional Interference Gender Identification | - | - |
| 21 | Lydon | 2015 | 18 | 32 | 28% | 12 | 2.6 | - | 12.6 | Incentivised Go/no-go | 0.18 | 0.05 |
| 22 | Mitchell | 2004 | 11 | 20 | 45% | 24 | 5.0 | 15 | 23.4 |  |  |  |
| 23 | Mendrek | 2006 | 15 | 35 | 40% | 13 | 5.1 | 15 | 21.9 | N-back | 0.59 | 0.08 |
| 24 | Merritt | 2010 | 25 | - | 52% | 24 | - | - | 14.6 | Digit Span | 0.09 | 0.04 |
|  |  |  |  |  |  |  |  |  |  | Recognition Memory | 0.38 | 0.04 |
|  |  |  |  |  |  |  |  |  |  | Spatial Orienting | <0.01 | 0.02 |
| 25 | Merritt | 2012 | 25 | 23 | 52% | 24 | 4.4 | - | 14 | Recognition Memory | 0.43 | 0.04 |
| 26 | Mogg | 2002 | 27 | 33 | 51% | 12 | 3.9 | 10 | - | Dot Probe | 0.01 | 0.04 |
|  |  |  |  |  |  |  |  |  |  | Smoking Stroop | <0.01 | 0.04 |
|  |  |  |  |  |  |  |  |  |  | Stroop | 0.02 | 0.04 |
| 27 | Munafò | 2003 | 43 | 28 | 51% | 24 | 2.2 | 1 | 17.8 | Smoking Stroop | 0.39 | 0.09 |
| 28 | Oliver | 2012 | 70 | 37 | 41% | 12 | - | 10 | 35.5 | Odd-one Out | 0.18 | 0.06 |
| 29 | Parrott | 1991 | 20 | - | 40% | 12 | - | 15 | - | Letter Cancellation | 0.33 | 0.04 |
| 30 | Parrott | 1998 | 30 | 25 | 53% | 12 | - | 10 | - | Letter Cancellation | 0.36 | 0.04 |
|  | **Name** | **Year** | **N** | **Mean Age (yrs)** | **Sex (% females)** | **Abstinence Period (hrs)** | **Mean FTND** | **Minimum Cig/ Day** | **Mean Baseline CO (ppm)** | **Cognitive Tasks** | **Effect Size d*** | **Variance** |
|  |  |  |  |  |  |  |  |  |  | Mental Arithmetic | 0.22 | 0.13 |
| 31 | Pomerleau | 1994 | 13 | 28 | 100% | 12 | 5.9 | 15 | - | Stroop | 0.66 | 0.08 |
| 32 | Powell | 2002 | 26 | 24 | 50% | 10 | 3.7 | - | 20 | CARROT | 0.54 | 0.04 |
|  |  |  |  |  |  |  |  |  |  | Saccade/antisaccade Eye Movement | - | - |
|  |  |  |  |  |  |  |  |  |  | Verbal Fluency | 0.20 | 0.04 |
|  |  |  |  |  |  |  |  |  |  | Reversed Digit Span | - | - |
| 33 | Powell | 2002 | 21 | 23 | 57% | 12 | - | 10 | 18.2 | Emotional Stroop | -0.84 | 0.07 |
| 34 | Roewer | 2015 | 37 | 33 | 41% | 24 | 7.2 | 25 | 16.1 | Delay Discounting | 0.25 | 0.03 |
| 35 | Roth | 1992 | 20 | - | 100% | 8 | - | - | 27 | Austin Maze |  | - |
|  |  |  |  |  |  |  |  |  |  | Word Recognition |  | - |
| 36 | Rusted | 1998 | 15 | 25 | 47% | 8 | 4.0 | - | 14.8 | Progressive Ratio | 0.71 | 0.08 |
|  |  |  |  |  |  |  |  |  |  | Rapid Visual Information | 0.59 | 0.08 |
| 37 | Sayette | 1994 | 40 | 21 | 70% | 12 | - | - | - | Cue Exposure Task | -0.17 | 0.06 |
| 38 | Shirtcliff | 2003 | 26 | - | - | 96 | - | 15 | - | Cued Target Detection | - | - |
| 39 | Soar | 2008 | 40 | 28 | 50% | 12 | - | - | - | Auditory Verbal Learning | 0.15 | 0.03 |
| 40 | Waters | 2000 | 24 | - | - | 24 | - | 7 | - | Smoking Stroop | -0.90 | 0.21 |
| 41 | Yi | 2012 | 28 | 40 | 28% | 24 | 6.4 | 20 | 19.4 | Delay Discounting | 0.65 | 0.04 |
| 42 | Zvolensky | 2014 | 258 | 44 | 30% | 16 | 5.2 | 10 | - | Relapse Analogue | 0.77 | <0.01 |

*a positive value of d indicates an effect in the expected direction (i.e., worse cognitive performance, higher attentional bias, or higher impulsivity in abstinent as compared with satiated smokers).
