# Supplementary material for: Behavioural tasks sensitive to acute abstinence and predictive of smoking cessation success: a systematic review and meta‐analysis
Source: Addiction. 2016 Aug 8;111(12):2134–44. doi: 10.1111/add.13507 (PMC5111768; doi:10.1111/add.13507)
Supplement: Supplementary file 2 — Table S2 Search 2 study characteristics. [file ADD-111-2134-s002.docx]

**Supplementary Table 2. Search 2 study characteristics**

|  |  | **Year** | **N Baseline** | **Mean Age** | **Sex**  **(% f)** | **Min Cig/ Day** | **Deprivation Baseline**** |  | **Challenges Included*** | **Follow- Up (months)** | **Cognitive Tasks** | **Effect Size**** |
| --- | --- | --- | --- | --- | --- | --- | --- | --- | --- | --- | --- | --- |
| 1 | Almeida | 2011 | 228 | 74 | 47% | 10 | s |  | Ph, Ps | 6, 12, 24 | Logical Memory | d = 0.03 |
| 2 | Brown | 2009 | 81 | 42 | 48% | 10 | s |  | none | 1 | Paced Auditory Serial Addition | RR=1 |
| 3 | Ditre | 2012 | 72 | 43 | 60% | 20 | a |  | Ph | 2, 5 | Startle Response | - |
| 4 | Goto | 2009 | 689 | 35 | 45% | - | a |  | none | 1, 2, 3, 4, 5 | Discrete Choice | HR= 1.17 |
| 5 | Hendricks | 2014 | 11 | 46 | 45% | 10 | a |  | Ph, Ps | 3 | Rapid Visual Information Processing | - |
| 6 | Kassel | 2007 | 28 | 49 | 58% | 10 | s |  | Ph, Ps | 12 | Emotional Stroop | d=1.12 |
| 7 | Lopez-Torrecillas | 2014 | 140 | 47 | 61% | - |  |  | Ph, Ps | 3, 6, 12 | Go/no-go | - |
|  |  |  |  |  |  |  | s |  |  |  | Delay Discounting | - |
|  |  |  |  |  |  |  |  |  |  |  | Iowa Gambling | - |
| 8 | Postma | 2001 | 18 | 29 | 72% | - | s |  | none | 1 | Startle Response | d=1.12 |
| 9 | Powell | 2010 | 141 | 33 | 54% | 10 |  |  | none | 1, 3 | Antisaccade | - |
|  |  |  |  |  |  |  |  |  |  |  | CARROT | - |
|  |  |  |  |  |  |  | s |  |  |  | Cue Reactivity | - |
|  |  |  |  |  |  |  |  |  |  |  | Motor Task | OR=1.09 |
|  |  |  |  |  |  |  |  |  |  |  | Stroop | - |
| 10 | Schlam | 2011 | 365 | 44 | 57% | 10 | s |  | Ph | 2 | Simon | d=0.29 |
| 11 | Sheffer | 2012 | 97 | 48 | 59% | 16 |  |  | none | 1, 2, 3, 7 | Balloon Analogue Risk | - |
|  |  |  |  |  |  |  |  |  |  |  | Delay Discounting | OR=0.62 |
|  |  |  |  |  |  |  | s |  |  |  | Go/no-go | - |
|  |  |  |  |  |  |  |  |  |  |  | Microcognitive Assesment | - |
|  |  |  |  |  |  |  |  |  |  |  | Stanford Time Perception | - |
| 12 | Sheffer | 2014 | 131 | 47 | 53% | 10 | s |  | Ph, Ps | 1, 2, 3, 4, 5, 6 | Delay Discounting | HR=1.49 |
| 13 | Secades-Villa | 2014 | 80 | 39 | 66% | 10 | s |  | Ps | 12 | Delay Discounting | d=0.14 |

*Ps = psychological challenges, Ph = pharmacological challenges; **d = Cohen’s d, RR = relative risk, HR = hazard ratio, OR = odds ratio; ** a = abstinent at baseline, s = satiated at baseline
